# Disease of the Antrum

**Published:** 1871-09

**Authors:** H. D. Boyd


### ARTICLE III.

### *Disease of the Antrum.*

By Dr. H. D. Boyd.

Sept. 1st, '71, Annie Y— age 30, called on me to have a tooth extracted. On examination I found the mouth generally, in a wretched condition, all of the upper teeth decayed near by to the gums, but not entirely gone, face and head giving general pain, right cheek slightly swollen, the right eye much protruded, the pupil presenting a pointed and decidedly greenish appearance, and the sight entirely suspended. General bad health for four months past, about which time the trouble commenced with the eye. I decided that the trouble was the result of disease of the antrum, and caused by the right superior first molar which I extracted in company with every other tooth or parts of teeth in the upper mouth, using ether as an anæsthetic. The patient went away, to call next day, but twelve hours after I was sent for, and found she had lost more than a quart of blood and was very uneasy. I arrested the hemorrhage easily by using ferri sub. sulph. Saw the patient three months after in good health, and with the eye in a normal condition.

---

